# Downstream of hearing loss: a population-based multistate analysis of lifetime risk and years lived with hearing loss, dementia and their comorbidity in Finland

**DOI:** 10.1093/ageing/afaf361

**Published:** 2025-12-19

**Authors:** Donata Stonkute, Angelo Lorenti, Kaarina Korhonen, Pekka Martikainen, Mikko Myrskylä

**Affiliations:** Max-Planck-Institute for Demographic Research, Rostock, Germany; Max Planck – University of Helsinki Center for Social Inequalities in Population Health, Helsinki, Finland; Max-Planck-Institute for Demographic Research, Rostock, Germany; Max Planck – University of Helsinki Center for Social Inequalities in Population Health, Helsinki, Finland; Max Planck – University of Helsinki Center for Social Inequalities in Population Health, Helsinki, Finland; University of Helsinki Helsinki Institute for Demography and Population Health, Helsinki, Uusimaa, Finland; Max-Planck-Institute for Demographic Research, Rostock, Germany; Max Planck – University of Helsinki Center for Social Inequalities in Population Health, Helsinki, Finland; University of Helsinki Helsinki Institute for Demography and Population Health, Helsinki, Uusimaa, Finland; Max-Planck-Institute for Demographic Research, Rostock, Germany; Max Planck – University of Helsinki Center for Social Inequalities in Population Health, Helsinki, Finland; University of Helsinki Helsinki Institute for Demography and Population Health, Helsinki, Uusimaa, Finland

**Keywords:** Hearing loss, Dementia, Lifetime risk, Multistate models, Health expectancy, Older people

## Abstract

**Background:**

Hearing loss (HL) is a major modifiable risk factor of dementia, yet gaps persist regarding its association with years lived with dementia, lifetime risk differences by HL status, and sociodemographic disparities.

**Methods:**

Using Finnish register data on all residents aged 60–99 from 2009–2019 (N=1,987,876; 16,439,107 person-years) and discrete-time multistate modeling, we calculated age-specific transition probabilities between five states – healthy, HL, dementia, comorbidity (both HL and dementia), and death, stratified by sex and education. We then estimated state-specific expectancies and lifetime risk.

**Results:**

At age 60, males and females in the overall population were expected to live 3.39 and 3.61 years with HL, and 1.09 and 1.85 years with dementia, respectively. Lifetime risk of HL was 22.7% for both sexes; dementia risk was 21.4% for males and 31.0% for females. When examining subpopulations defined by origin state at age 60, those who were healthy could expect 1.10 (males) and 1.89 (females) years with dementia. Meanwhile, those with HL at age 60: 1.90 years (males) and 2.82 years (females) with both HL and dementia (comorbidity). Their lifetime risk of comorbidity was 33.5% (males) and 42.9% (females) – about 1.5 times higher than the dementia risk for those starting healthy. Higher education was associated with longer life, more years across all states, and higher lifetime risks.

**Conclusions:**

These findings illustrate how HL at age 60 is associated with a redistribution of remaining years toward dementia, providing new insight into the joint burden of sensory and cognitive aging.

## Key Points

- Individuals with HL at age 60 live approximately one additional year with dementia compared to healthy individuals.
- The lifetime risk of dementia is about 1.5 times higher for individuals with HL at age 60.
- Female sex, higher levels of education are associated with more morbid years due to longer exposure to age-related diseases.
- Early detection and management of hearing loss may help reduce the burden of dementia.

## Introduction

Dementia represents a significant global health challenge, with the number of people living with dementia projected to increase to 153 million by 2050 [1]. It is among the leading causes to disability burden [2] and has far-reaching social and economic consequences for families and societies at large [3]. Age-related hearing loss (HL) is another prevalent condition in aging populations [4], with well-documented associations with social withdrawal, loneliness, and depression [5, 6]. Notably, there is increasing evidence linking HL to cognitive decline [7] and dementia [8].

HL is associated with cognitive decline across multiple cognitive domains, as well as an increased risk of cognitive impairment and dementia incidence [9]. Maharani *et al.* [10] found that older adults with hearing impairment had lower episodic memory scores and were at a greater risk of cognitive impairment. Similarly, Matthews *et al.* [11] demonstrated that individuals who consistently experienced poorer or worsening hearing had a more rapid cognitive decline. Impaired hearing is also linked to neurodegeneration relevant to dementia [12].

Differences by sex/gender exist for both health outcomes. HL disproportionately affects men [13, 14], while women have a higher lifetime risk of dementia [15]. Social inequalities also play a critical role in shaping the landscape of both HL and dementia. Individuals with lower educational attainment are more susceptible to both HL and dementia [16, 17].

Despite extensive literature documenting HL as a modifiable risk factor of dementia, three critical knowledge gaps remain unaddressed. First, current health expectancy research has focused primarily on estimates for HL [18, 19] and dementia [20, 21] as individual conditions, leaving unclear how many years individuals live with comorbidity – that is having both HL and dementia. Second, despite the importance of understanding lifetime risk – the probability an individual will develop a condition during their lifespan – current research lacks comparative estimates of dementia lifetime risk between individuals with and without HL. Third, while both conditions independently vary by education and sex, we have limited insight into how these sociodemographic factors may compound vulnerabilities in the HL-dementia associations. To address these gaps, our study leverages Finnish population-level register data to provide comprehensive estimates of years lived with HL, dementia, and their comorbidity, as well as lifetime risks, by sex and education – revealing critical insights for identifying increased-risk populations and informing targeted interventions.

## Data and methods

### Data and study population

This study used population register data covering all permanent residents in Finland. We restricted individuals to those aged 60-99 during years 2009-2019. The restriction is due to the age pattern of health outcomes considered. We linked annual population register information with the Death Register for dates of death using personal identification codes assigned to all permanent residents. Similarly, we integrated population register information with data from various national health records: Finnish Drug Reimbursement Register (available from 1995, though anti-dementia medications only appear from 2000 onward), which included medication purchases and reimbursements, provided by the Social Insurance Institution of Finland, as well as the Care Register for Health Care, from which we utilized hospitalization data (1987-2019) and specialized care visits (1998-2019), maintained by the National Institute for Health and Welfare. To be included in the multistate calculation, individuals needed to contribute to person-years in at least two calendar years. Following exclusions, the final analytical sample comprised 1,987,876 individuals, contributing to 16,439,107 person-years. See Appendix S1 in the Supplementary Data section for the full details of the analytical sample derivation process.

### Dementia

The Drug Reimbursement and Care registries were used to determine the year of dementia onset. Dementia was identified based on several criteria, including eligibility for reimbursement of Alzheimer’s disease medication (disease code 307), the purchase of state-reimbursed Alzheimer’s disease medication (Anatomical Therapeutic Chemical [ATC] code N06D), and records from inpatient and specialized outpatient care registries where dementia was listed as a primary or additional diagnosis. Diagnoses were classified according to the International Classification of Diseases, 9th Revision (ICD-9) for the years 1987–1995 (codes 290, 291.2, 292.8C, 294.1A, 331.0, 331.1, and 437.8A) and the 10th Revision (ICD-10) for the years 1996–2019 (codes F00–F03, F05.1, and G30). The use of a combination of Finnish hospital care and medication reimbursement registers to identify cases of dementia has previously been validated [22] and employed in empirical investigations [23]. The year of dementia onset was determined by identifying the earliest recorded occurrence in these registries for individual persons.

### Hearing loss

Hearing loss (HL) was identified using data from inpatient and specialized outpatient Care registries for the years 1996-2019. Identification was based on either a documented diagnosis of conductive and sensorineural HL (ICD-10 code H90), other and unspecified HL (ICD-10 code H91), or evidence of hearing aid (HA) use, including fitting and adjustments of devices (ICD-10 codes Z45.3, Z46.1, Z97.4). This coding strategy optimizes both etiological comprehensiveness, by capturing a wide range of HL pathophysiology, and sensitivity, by supplementing clinical diagnoses with HA utilization codes to identify functionally significant cases that might otherwise be missed in register data. The earliest recorded occurrence was considered the year of HL onset.

### Sociodemographic variables

Information on sex, age, and educational attainment was provided in the population register of Statistics Finland. Age was a continuous variable measured in completed years. Educational attainment was determined based on the highest qualification achieved and classified according to the International Standard Classification of Education (ISCED-2011) as follows: tertiary (levels 5–8), secondary (levels 3–4), and no qualifications (levels 0–2).

### Analytical approach

This study employs discrete-time Markov chain multistate models analyzing transitions across five states: healthy, HL, dementia, comorbidity, and death. Health states are defined by the presence or absence of HL and dementia; thus, the `healthy' state denotes the absence of diagnoses for both conditions, rather than overall health. The `comorbid' state refers specifically to the co-occurrence of HL and dementia. Multistate models allow us to calculate years of remaining life at age 60 expected in these health states during 2009-2019, differentiate the mortality patterns associated with each health state, and estimate the lifetime risk of developing morbidity through various progression pathways (See Appendix S2 in the Supplementary Data section).

In Markov chain multistate models, the estimation of health expectancies and lifetime risks relies on transition probabilities, which quantify the likelihood of movement between health states. Age-specific transition probabilities were obtained non-parametrically from the empirical data by tabulating proportions of movements from origin to destination states and calculated for each age stratifying by sex and education. The use of full-population data allows for direct calculation of transition probabilities without relying on statistical modeling or distributional assumptions, thereby providing robust estimates based entirely on observed transitions.

The sex and education specific matrices were used to derive the corresponding fundamental matrices [24]. By means of the transition matrices and the corresponding fundamental matrices, we compute state-specific expectancies, conditioned and unconditioned on starting state, and life time risks, conditioned on starting state.

State expectancies are a weighted sum of conditional expected durations in a given state. To account for the mid-period transition assumption, a half-year correction of 0.5 was subtracted from the sum of transitions between states before applying the starting distribution weights. To mitigate noise from small sample sizes, starting distribution weights were calculated using a 5-year age interval of 60-65 over the period 2009-2019. Lifetime risk was calculated as a cumulative probability of ever transitioning into a specific state from a given state of origin. More technical details are available in existing literature [15, 25].

It is important to note that the study covers a 10-year period (2009–2019), thus the estimates are based on synthetic cohorts constructed from observed age-specific transition probabilities. These estimates therefore reflect the experiences of individuals aged 60 and older during the study period.

Confidence intervals were obtained using a non-parametric bootstrap approach [26]. Due to computational constraints with the full population, we generated 200 resamples from a 5% random subsample by resampling individual trajectories, estimated the length of the confidence intervals based on this subsample, and obtained the length of the total population confidence intervals by scaling the 5% sub-sample by factor of 1/sqrt(20), as the total population is 20 times the 5% sub-sample. We constructed the confidence intervals by applying the length symmetrically around the point estimate.

Data management and preparation were conducted using Stata version 18.0, while all statistical analyses were implemented in R version 4.4.0.

## Results

### Descriptive statistics

The total sample size was 905,394 males and 1,082,482 females, and the analysis was based on 7,332,239 and 9,106,868 person-years, respectively (Table 1). A total of 460,482 deaths were observed during the study period. On average, females and those with no educational qualifications were represented by older ages.

**Table 1:** Sample characteristics of adults aged 60-99 in Finland upon their entry into the sample, 2009-2019 by sex and educational attainment level

	**Males**				**Females**			
	**No qualifications**	**Secondary**	**Tertiary**	**Total**	**No qualifications**	**Secondary**	**Tertiary**	**Total**
**Age, mean**	68.7	63.4	64.5	65.9	72.1	64.9	64.1	67.9
**State, N (%)**								
Healthy	333,763 (86.9)	263,997 (91.7)	213,780 (91.5)	811,540 (89.6)	415,820 (84.2)	303,828 (91.1)	234,005 (92.0)	953,653 (88.1)
Hearing Loss	33,159 (8.6)	19,269 (6.7)	15,646 (6.7)	68,074 (7.5)	39,924 (8.1)	21,356 (6.4)	15,539 (6.1)	76,817 (7.1)
Dementia	14,089 (3.7)	3,824 (1.3)	3,457 (1.5)	21,370 (2.4)	32,861 (6.7)	7,633 (2.3)	4,184 (1.6)	44,678 (4.1)
Comorbid	3,004 (0.8)	742 (0.3)	664 (0.3)	4,410 (0.5)	5,528 (1.1)	1,124 (0.3)	682 (0.3)	7,334 (0.7)
Total	384,015 (*42.4*)	287,832 (*31.8*)	233,547 (*25.8*)	905,394 (100)	494,133 (*45.7*)	333,941 (*30.9*)	254,408 (*23.5*)	1,082,482 (100)

*Note*: Percentages are to be interpreted as column-wise percentages, with the exception of the final row Total, which provides educational composition for males and females.

**Figure 1:**
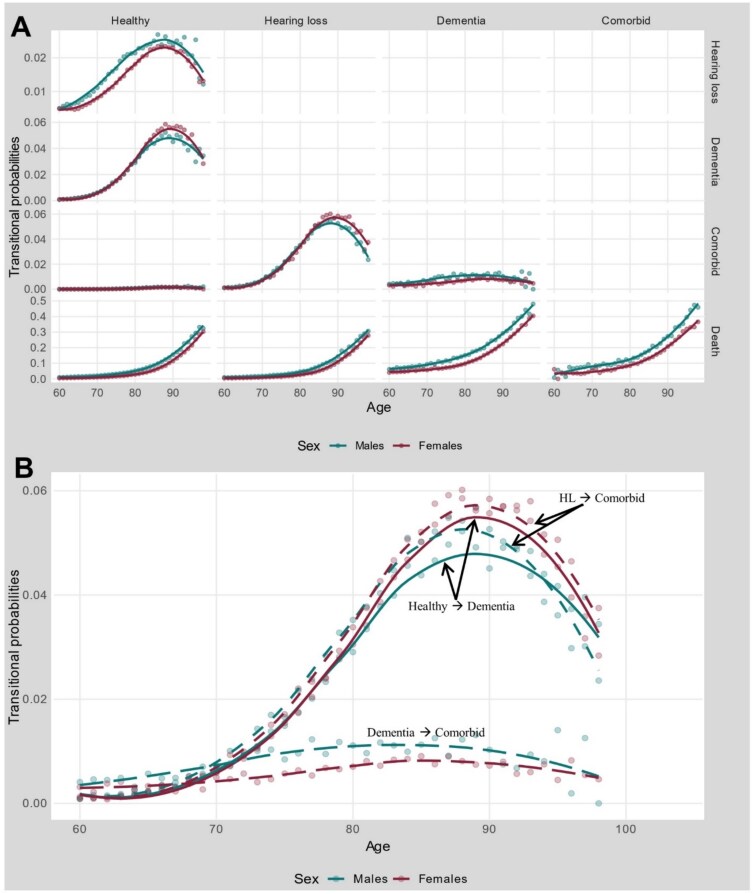
Sex-specific transition probabilities. **Panel A** presents sex-specific transition probabilities across age from origin states to destination states, excluding transitions within the same state. **Panel B** compares selected transition probabilities by sex. *Source:* Author’s calculations based on population register data of all Finns aged 60–99 from 2009 to 2019. *Note:* Smoothing lines were applied using the LOESS method for readability, while multistate life tables were constructed using transitional probabilities, derived from observed transitions across single-year age groups.

Mortality risk increased exponentially with age, exhibiting notable sex differences (Figure 1). Males consistently demonstrated higher death transition probabilities regardless of origin state. Individuals with dementia or comorbidity exhibited substantially higher mortality than those healthy or with HL alone, though HL did not increase mortality risk compared to healthy individuals.

Sex-specific patterns emerged across health transitions. Females showed higher probabilities of transitioning from healthy to dementia and from HL to comorbidity, with differences pronounced after age 80. Males showed higher probabilities of transitioning from healthy to HL and from dementia to comorbidity, with consistent differences across all ages. For both sexes, transition probabilities into comorbidity were consistently higher among individuals with pre-existing HL compared to those healthy or with pre-existing dementia (Figure 1).

Patterns across educational attainment levels were less consistent. See Appendix S3 in the Supplementary Data section for the full details of education-specific estimates.

### Life and health expectancies

Remaining life expectancy at age 60 was 21.40 for males and 25.41 for females, reflecting a female survival advantage (Table 2). The figures are consistent with Human Mortality Database [27] for 2010-2019, which reported 21.85 for males and 25.94 for females. This deviation is to be expected because our study was restricted to age 99.

**Table 2:** Overall and state-specific life expectancies (LE) at age 60, and LE by starting state (healthy, hearing loss (HL), dementia, or comorbidity), by sex and educational level in Finland (2009–2019), with 95% confidence intervals

	Population-level		By education					
	**Males**	**Females**	**Males**			**Females**		
			No qualifications	Secondary	Tertiary	No qualifications	Secondary	Tertiary
**Life and Health Expectancies** *Unconditional on starting state*								
Overall LE	21.40 [21.37-21.43]	25.41 [25.38-25.44]	20.20 [20.15-20.25]	21.48 [21.42-21.54]	23.71 [23.63-23.79]	24.56 [24.51-24.61]	25.76 [25.69-25.83]	27.04 [26.95-27.13]
Healthy LE	16.39 [16.35-16.43]	19.25 [19.22-19.28]	15.66 [15.61-15.71]	16.14 [16.08-16.20]	18.10 [18.04-18.16]	18.84 [18.79-18.89]	19.33 [19.27-19.39]	20.27 [20.19-20.35]
LE with HL	3.39 [3.36-3.42]	3.61 [3.58-3.64]	3.00 [2.97-3.03]	3.70 [3.65-3.75]	3.81 [3.75-3.87]	3.18 [3.14-3.22]	3.83 [3.78-3.88]	4.17 [4.10-4.24]
LE with dementia	1.09 [1.08-1.10]	1.85 [1.84-1.86]	1.06 [1.05-1.07]	1.09 [1.06-1.12]	1.21 [1.18-1.24]	1.88 [1.86-1.90]	1.85 [1.82-1.88]	1.89 [1.85-1.93]
LE with Comorbidity	0.53 [0.52-0.54]	0.71 [0.70-0.72]	0.48 [0.47-0.49]	0.56 [0.54-0.58]	0.59 [0.56-0.62]	0.67 [0.66-0.78]	0.75 [0.73-0.77]	0.71 [0.68-0.74]
**Life expectancy** *Conditional on starting state at age 60*								
Healthy	21.43 [21.40-21.46]	25.46 [25.43-25.49]	20.25 [20.20-20.30]	21.50 [21.43-21.57]	23.74 [23.66-23.82]	24.63 [24.58-24.68]	25.81 [25.75-25.87]	27.08 [26.99-27.17]
HL	22.19 [22.09-22.29]	25.85 [25.75-25.95]	21.06 [20.90-21.22]	22.41 [22.21-22.61]	24.19 [23.99-24.39]	25.00 [24.82-25.18]	26.11 [25.93-26.29]	27.35 [27.15-27.55]
Dementia	10.67 [10.41-10.93]	14.13 [13.86-14.41]	10.23 [9.82-10.63]	10.72 [10.32-11.13]	11.61 [11.05-12.16]	13.72 [13.27-14.18]	14.06 [13.60-14.52]	14.99 [14.36-15.62]
Comorbidity	12.82 [12.30-13.34]	16.07 [13.92-18.23]	12.08 [9.94-14.22]	13.13 [11.18-15.09]	13.47 [11.22-15.72]	15.45 [12.88-18.01]	15.84 [15.84-15.84]	17.84 [17.29-18.40]

*Note*: All expectancies are in years. Life expectancies conditional on a starting state are based on trajectories of subpopulations defined by the state they occupy at age 60.

Remaining years of life were partitioned into expected time spent in each of the four health states. At age 60, males could expect to spend 16.39 years without either of the conditions, 3.39 with HL, 1.09 with dementia and 0.53 with comorbidity. Females at the same age could expect to spend 19.25 years healthy, 3.61 with HL, 1.85 with dementia, and 0.71 with comorbidity.

The longer life expectancy of females was accompanied by longer healthy life, as well as, more years lived with HL, dementia, and their co-occurrence, compared to males. Education was also associated with both longer healthy life expectancy and more time spent in morbid states. Specifically, individuals with tertiary education, when compared to peers with no educational qualifications, demonstrated longer life expectancy: 3.51 years for males and 2.48 for females, accompanied by more time spent with HL, dementia, or both by approximately one year for each sex.

Expected years lived with dementia differed markedly by health status at age 60. Specifically, these are conditional expectancies representing the number of years an individual can expect to live given their health state at age 60. Males with HL at age 60 could expect 22.19 years on average (Table 2), of which 1.90 would be spent with co-occurring HL and dementia (Table 3). Females could expect 25.85 years on average, of which 2.82 would be spent with co-occurring HL and dementia. Those who were healthy (free from HL and dementia) at age 60 had a similar total life expectancy (21.43 years for males and 25.46 years for females), but approximately one year less with dementia: 1.10 years for males and 1.89 years for females. A complete list of conditional expectancies can be found in Appendix S4 in the Supplementary Data section.

**Table 3:** Remaining years in healthy state, with hearing loss (HL), dementia, or comorbidity for two subpopulations: Starting healthy or with HL at age 60, with corresponding 95% confidence intervals

**Remaining years in destination states:**	**Males**		**Females**	
	**Starting Healthy** (92.5%)	**Starting with HL** (5.8%)	**Starting Healthy** (93.2%)	**Starting with HL (**5.7%)
Healthy	17.52 [17.49-17.55]	-	20.53 [20.50-20.56]	-
HL	2.37 [2.35-2.39]	20.29 [20.20-20.38]	2.46 [2.44-2.48]	23.03 [22.93-23.13]
Dementia	1.10 [1.08-1.11]	-	1.89 [1.87-1.90]	-
Comorbidity	0.44 [0.43-0.44]	1.90 [1.86-1.93]	0.58 [0.57-0.58]	2.82 [2.77-2.86]

*Note*: the numbers in brackets (%) indicate the proportion of each subpopulation aged 60–65 within the full population – i.e., their prevalence. These proportions, along with those for dementia and comorbidity states at age 60–65, were used as weights to calculate population averages.

Empty cells represent impossible transitions, as HL and dementia are modelled as mutually exclusive and irreversible states (Appendix 2 in the Supplementary Data section). Transitions from these states are only possible toward comorbidity.

### Lifetime risk

**Figure 2:**
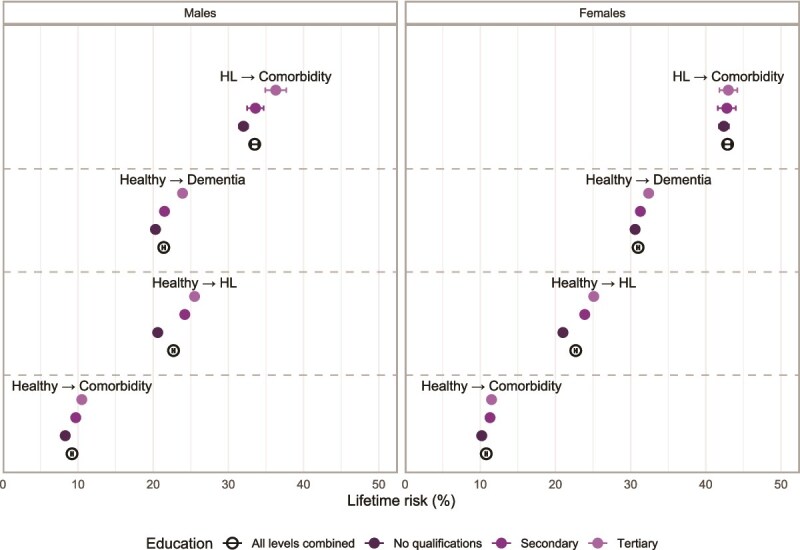
Lifetime risks by sex and education. *Points represent the estimated lifetime risk (%) of transitioning from the given origin state (e.g., Healthy or HL) to another health state. Bars indicate 95% confidence intervals.*

The lifetime risk of developing HL from a healthy state was identical between sexes, despite females having lower age-specific incidence probability (22.7%, Figure 2). Partial risk between ages 60-80 was twice larger in males than females (16.5% vs. 8.3%, respectively), suggesting that females' longer survival allows them to accumulate similar lifetime risk despite lower age-specific incidence (see Appendix S5 in the Supplementary Data section 5). Furthermore, females exhibited substantially higher lifetime risk of developing dementia from a healthy state compared to males (21.4 for males vs. 31.0% for females, Figure 2). The lifetime risk of developing comorbidity directly from a healthy state was relatively low but still higher among females.

In addition to these sex-specific patterns, the health state at age 60 was also associated with differences in lifetime dementia risk. Among individuals starting out healthy at age 60, the lifetime risk of developing dementia was 21.4% for males and 31.0% for females. In contrast, for those already experiencing HL at age 60, the risk of transitioning to comorbid state was notably higher – 33.5% for males and 42.9% for females.

Finally, educational attainment revealed additional gradients in lifetime dementia risk. For males, the lifetime risk of developing dementia from a healthy state increased progressively with education, from 20.3% (no educational qualifications) to 23.9% (tertiary education). A similar pattern emerged for females, with dementia risk increasing from 30.6% to 32.4% across educational categories. Similar educational gradients were observed for other transitions, particularly for males, where the effect of education appears more pronounced than for females.

## Discussion

Hearing loss (HL) is increasingly recognized as a key target for dementia prevention, due to both its modifiability and prevalence in older adults [28]. However, we lacked clear understanding on whether and how the presence of HL should inform expectations about future years lived with dementia and the lifetime risk of dementia to support clinical decision-making. This study builds on existing knowledge by quantifying the association between HL and both years lived with comorbidity and lifetime risk of comorbidity, using full-population register data.

We find several important patterns. First, HL at age 60 is associated with approximately one additional year with dementia compared to those without HL, despite similar overall remaining life expectancy. Our finding on survival aligns with previous literature showing that HL does not reduce overall life expectancy [19, 29], despite its reported association with various adverse health outcomes [30, 31]. While West and Lynch [19] attributed this finding – which contradicts earlier literature linking HL to increased mortality risk [32] – to the possibility of recovery from hearing impairment in their survey-based data, our results emerge in a different context. We employ diagnosed HL, which relies on individuals recognizing difficulty of hearing and seeking healthcare. As such, it is more likely to capture persistent or non-recoverable HL compared to self-reported assessments – particularly at younger ages – where overestimation is common [33]. Both methodological approaches converge on the conclusion that HL is not associated with a reduction of remaining life years, with our findings specifically pointing that HL at age 60 is associated with a substantial redistribution of years toward increased time spent with dementia.

An additional explanation for the finding that HL (compared to healthy) and comorbidity (compared to dementia) do not reduce life expectancy may be a result of a selection bias: individuals with higher education are more likely to seek care and receive HAs, and therefore to be captured in registers. For example, recent evidence shows that associations between HA use and dementia are greatly reduced after adjusting for healthcare use, underscoring the role of healthcare utilization [34]. However, in our data the educational composition of those with HL closely resembles that of individuals without HL, making it unlikely that disproportionate healthcare use across educational groups alone could account for the observed patterns.

Second, we find that the higher dementia incidence associated with HL [35–37] comes with a substantially higher lifetime risk of developing dementia. This represents an approximate 12 percentage point increase, or 1.5 times higher, lifetime risk of dementia associated with HL at age 60, a clinically meaningful difference that persists across both sexes. This increase in lifetime risk is consistent with the evidence that HL is associated with cognitive decline and dementia pathology [10–12]. These estimates should also be interpreted against the broader evidence on dementia lifetime risk in the general population.

In the United States, the lifetime risk of dementia at age 55 has been estimated at 42% [38], while mortality-linked studies suggest that 20–39% of adults have dementia in the years preceding death [39]. Dutch registry data further show that 24% of people die with dementia, with proportions exceeding 40% among the oldest women [40]. These findings confirm that the magnitudes observed in our study are consistent with international evidence and underscore the high cumulative burden of dementia in aging populations.

It is also important to note that only about 6 percent of males and females at ages 60-65 in the Finnish population of this study were diagnosed with HL (as compared to 19 percent among males and 14 percent among females at ages 80-85), which likely reflects a subset with more severe or symptomatic impairments that prompt clinical attention. This early-onset (midlife) HL may therefore imply an early clinical marker of elevated neurodegenerative vulnerability rather than two isolated age-related morbidities. In line with this, Kim et al. [41] reported that the middle-aged individuals demonstrate higher risk of dementia associated with hearing impairment than the older-aged groups, underscoring greater significance of HL when it manifests at relatively younger ages.

Third, our findings reveal important sociodemographic patterns in health expectancy and lifetime risk. While females lived longer than males, their extended lifespan consisted of not only more healthy years but also more years with morbidities, including dementia. Female disadvantage extended to lifetime risks – females faced approximately 10 percentage points higher lifetime dementia risk regardless of HL status. This reflects the female-male health–survival paradox: women outlive men but spend greater proportions of their lives with disability and chronic illness, including dementia [42, 43].

Educational gradients were also evident: individuals with higher educational attainment experienced expanded morbid life expectancy and increased lifetime risks of HL, dementia, and their comorbidity, despite spending more years healthy. This reflects the longevity advantage of higher education, which increases cumulative exposure to age-related morbidities, consistent with evidence showing that longer lives among the more educated include more years with cognitive impairment [44]. The incidence-based life table framework we employed is especially useful here, as it reveals how the interaction of higher mortality among the less educated compresses their exposure to dementia over their lifetime, whereas the longer survival of the more educated expands it [45].

This study benefits from comprehensive Finnish register data covering all residents aged 60–99 and a 10-year follow-up, minimizing attrition and selection issues common in panel data. However, selection bias may persist. Diagnostic likelihood may vary by education, with higher-educated individuals more likely to seek care, potentially leading to underdiagnosis in lower-educated groups and underestimation of educational inequalities. Additionally, our outcome definition may have missed milder or undiagnosed dementia cases, potentially introducing differential misclassification by health-care utilization factors such as sex or education, although prior validation studies suggest high specificity [22]. Additionally, the temporal ordering between HL and dementia could not be fully established within our design, and the analyses did not account for vascular, metabolic, or psychosocial factors that may underlie both conditions.

Furthermore, our conceptual framework considered irreversible HL, yet some cases of HL may be reversible or mitigated through interventions. HA represent a common intervention, but their efficacy in reducing dementia risk remains contested. While some observational studies suggest potential cognitive benefits [46], consistent evidence supports HA advantages only among individuals already at high dementia risk [47, 48]. Our identification strategy, incorporating both HL diagnoses and HA utilization codes, enhances sensitivity to clinically relevant HL but complicates interpretation, as HA use may simultaneously indicate HL severity and potentially modify its effects on dementia.

In conclusion, HL is more than a sensory impairment – it is associated with patterns of cognitive aging and dementia risk. Our findings show that HL at age 60 is associated not only with more years with dementia but also a substantially higher lifetime risk of developing it. These burdens are not evenly distributed: females and those with higher education spend more years navigating both HL and dementia, reflecting the complex interplay of longevity and increased exposure to age-related conditions. Together, these findings have significant implications for clinical practice, patient care, and public health policy. Early identification and management of HL may therefore support public health efforts to promote cognitive health, though clear causal mechanisms remain to be established.

## Supplementary Material

Supplementary_materials_afaf361
