# Supplementary material for: Downstream of hearing loss: a population-based multistate analysis of lifetime risk and years lived with hearing loss, dementia and their comorbidity in Finland
Source: Age Ageing. 2025 Dec 19;54(12):afaf361. doi: 10.1093/ageing/afaf361 (PMC12716279; doi:10.1093/ageing/afaf361)
Supplement: Supplementary_materials_afaf361 [file supplementary_materials_afaf361.docx]

Individuals aged 60-99 between 2009-2019:

2,062,354 individuals

Dementia onset:

263,766 cases matched

Hearing loss onset:

299,649 cases matched

Death registry:

465,058 deaths matched

Excluding persons that contribute only 1 person-year (observed a single calendar year):

74,478

Final analytical sample:

1,987,876 individuals

460,482 deaths

16,439,107 person-years

**Appendix 1.** Flow chart of analytical sample derivation.


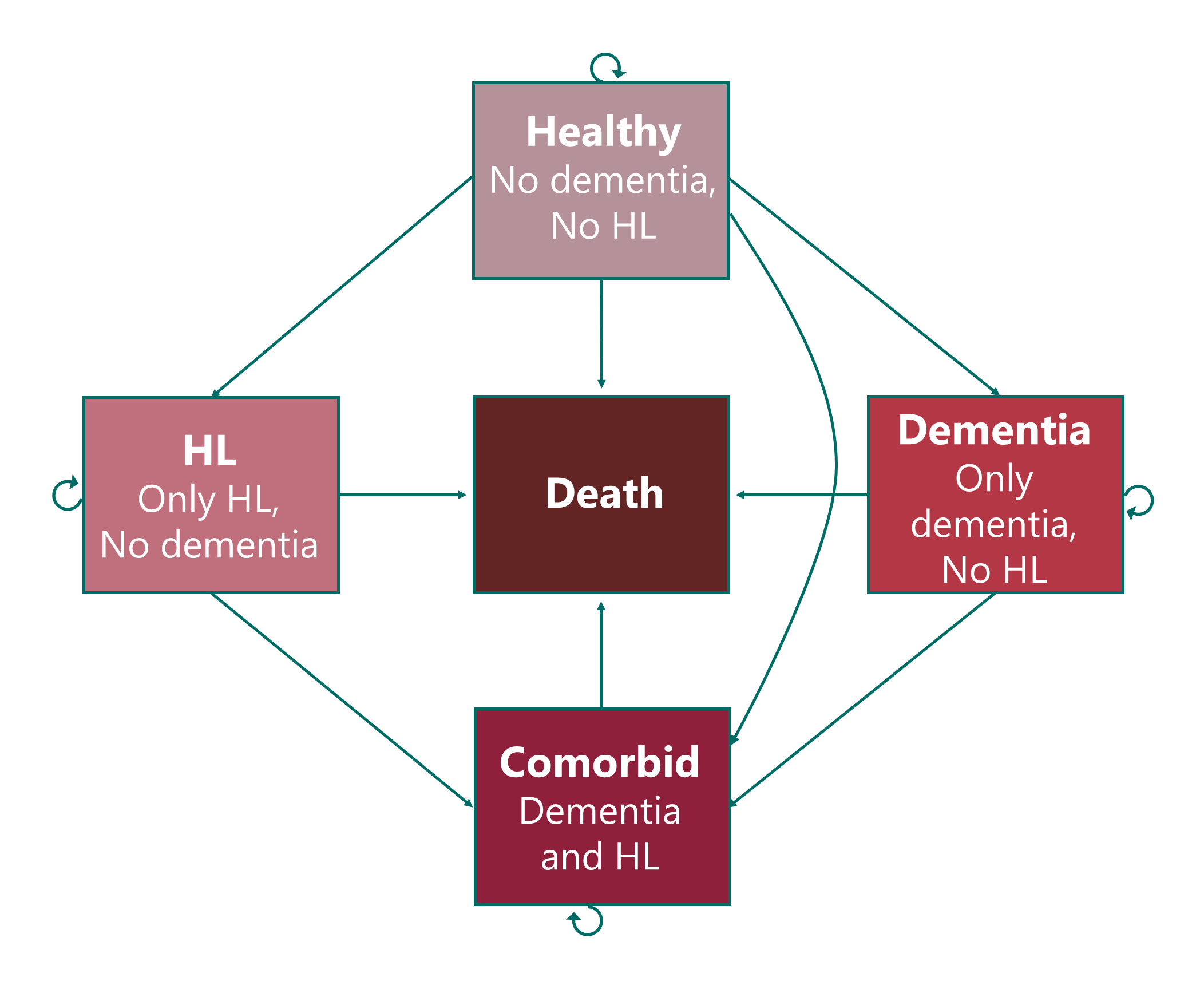


**Appendix 2.** State space of the Markov multistate model and possible transitions across the states.

*Note*: HL = hearing loss.

We calculated transition probabilities non-parametrically as tabulated proportions of movements between states across 1-year age groups, stratified by sex and education. Since the disease progression of hearing loss and dementia is irreversible, we define these transitions as non-recoverable.


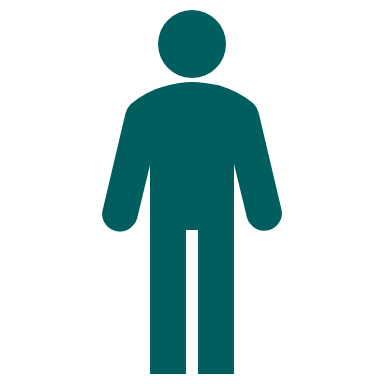

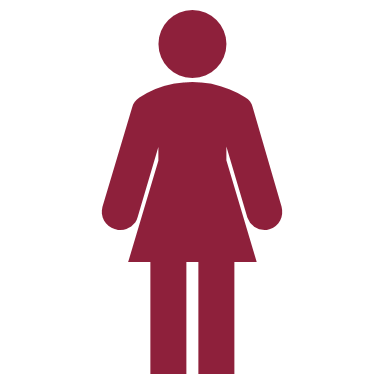


**Appendix 3.** Education-specific transition probabilities across age from origin states to destination states, excluding transitions within the same state, stratified by sex.
*Source:* Author’s calculations based on population register data of all Finns aged 60–99 from 2009 to 2019.
*Note:* Smoothing lines were applied using the LOESS method for readability, while multistate life tables were constructed using non-smoothed estimates. Age 99 is omitted from the graph for readability, as all individuals in this age group transition to the absorbing state of death.


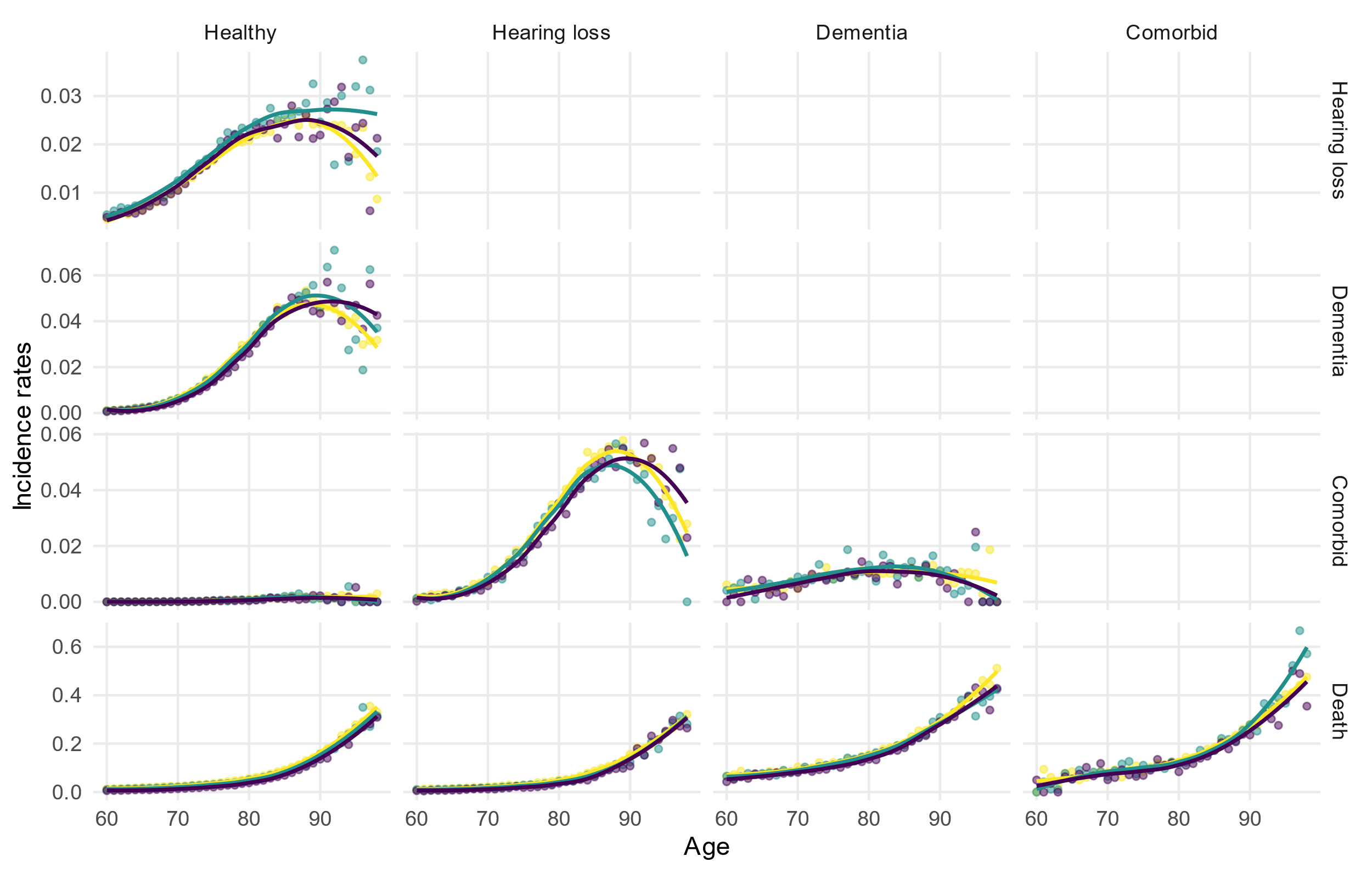

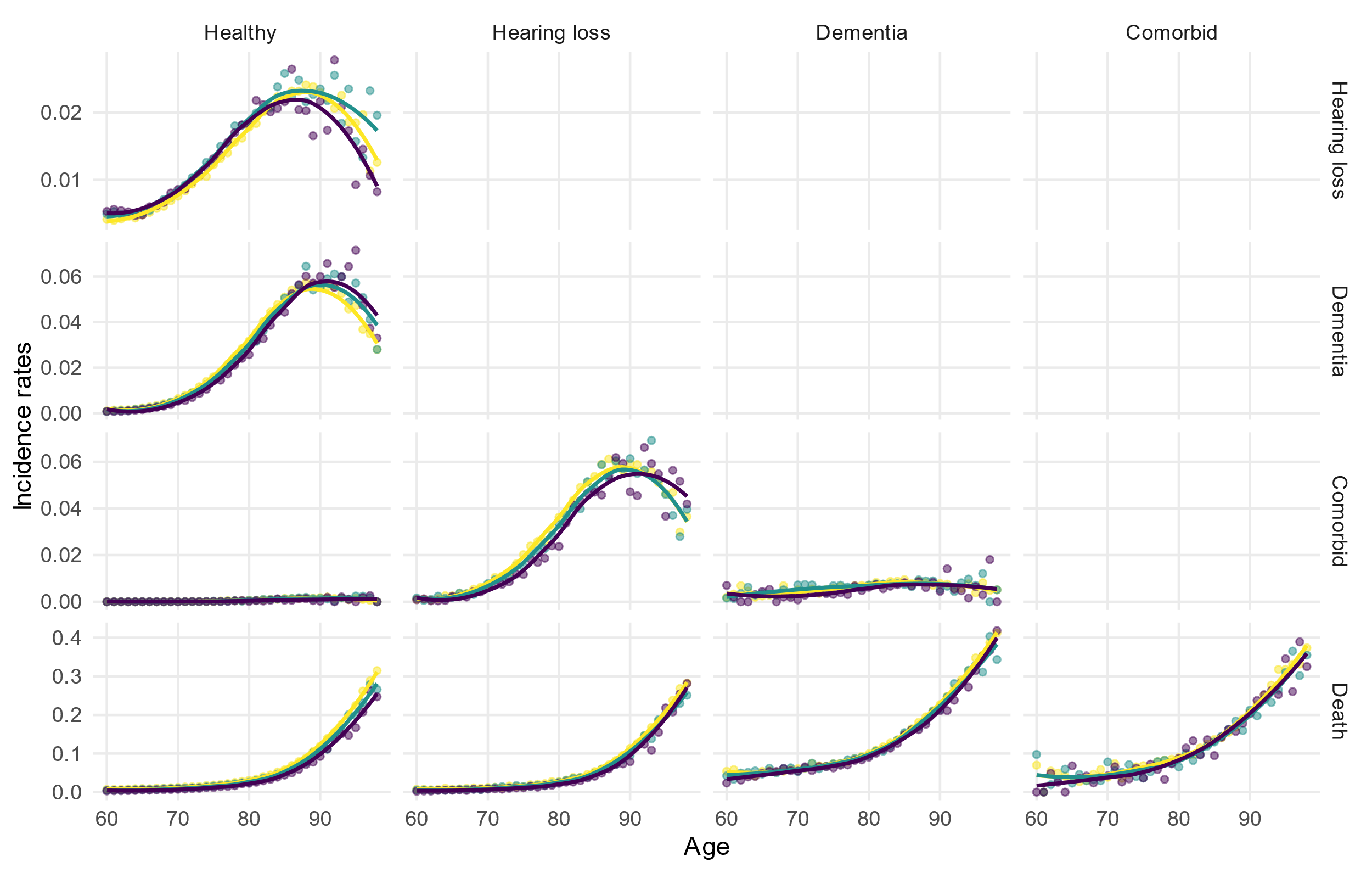

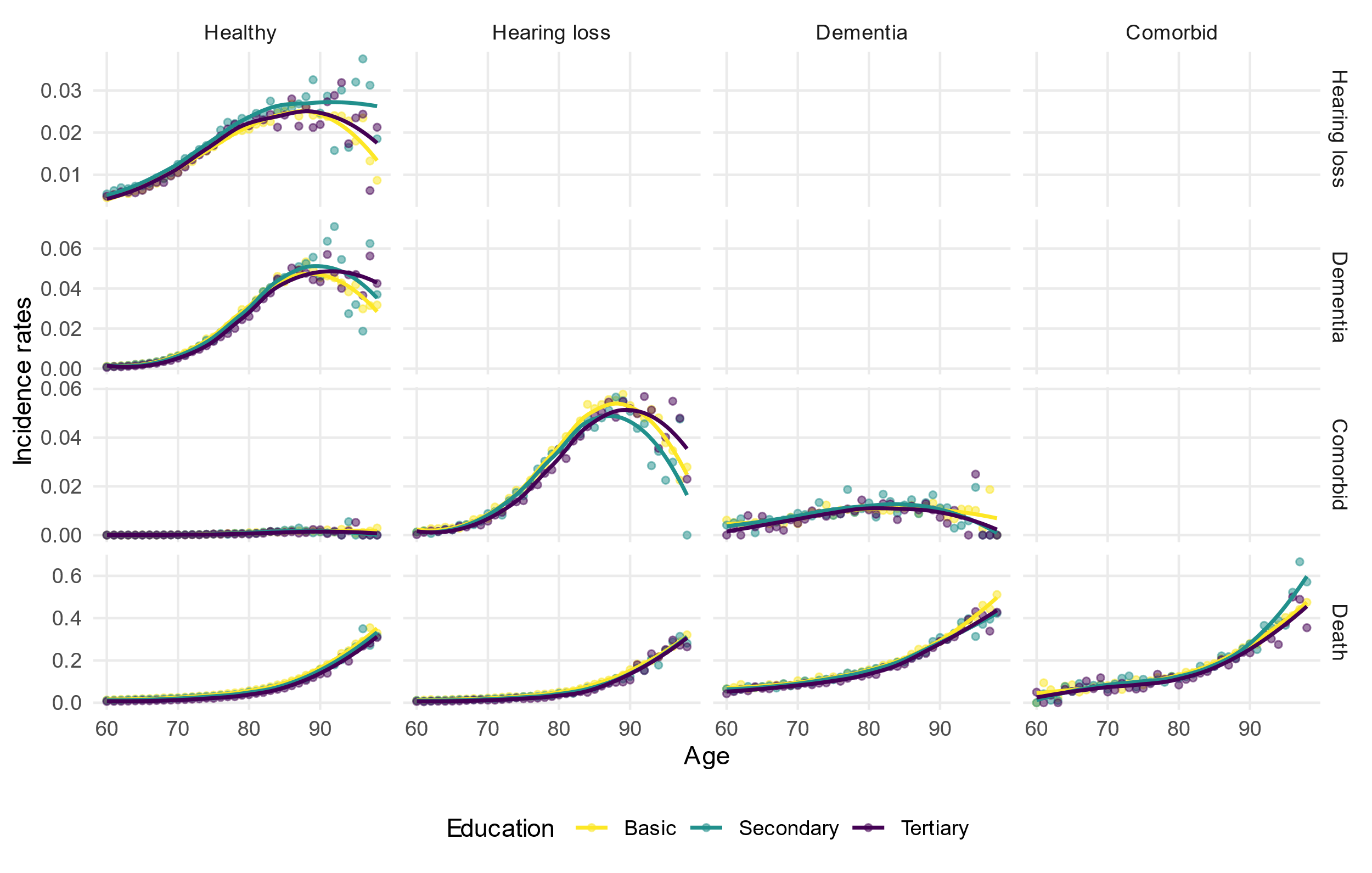


**Appendix 4.** Conditional Life and Health Expectancies.
To obtain the unconditional expectancies (AVERAGE), which do not depend on the initial state individuals start in, we computed weighted averages of the conditional expectancies. For each population stratum of interest, the weights were determined based on the starting state distribution at 60-65.

*A. Remaining Life Expectancy*

| Sex | Education | start.Healthy_60 | start.HL_60 | start.Dem_60 | start.Comorb_60 |
| --- | --- | --- | --- | --- | --- |
| Males | Population | 21.40 | 22.19 | 10.67 | 12.82 |
| Males | Basic | 20.25 | 21.06 | 10.23 | 12.08 |
| Males | Secondary | 21.50 | 22.41 | 10.72 | 13.13 |
| Males | Tertiary | 23.74 | 24.19 | 11.61 | 13.47 |
| Females | Population | 25.46 | 25.85 | 14.13 | 16.07 |
| Females | Basic | 24.63 | 25.00 | 13.72 | 15.45 |
| Females | Secondary | 25.81 | 26.11 | 14.06 | 15.84 |
| Females | Tertiary | 27.08 | 27.35 | 14.99 | 17.84 |

*B. Remaining Healthy Life Expectancy*

| Sex | Education | start.Healthy_60 |
| --- | --- | --- |
| Males | Population | 17.52 |
| Males | Basic | 16.72 |
| Males | Secondary | 17.34 |
| Males | Tertiary | 19.25 |
| Females | Population | 20.53 |
| Females | Basic | 19.99 |
| Females | Secondary | 20.68 |
| Females | Tertiary | 21.65 |

*C. Remaining Life Expectancy with Hearing Loss*

| Sex | Education | start.Healthy_60 | start.HL_60 |
| --- | --- | --- | --- |
| Males | Population | 2.37 | 20.29 |
| Males | Basic | 2.08 | 19.22 |
| Males | Secondary | 2.60 | 20.52 |
| Males | Tertiary | 2.76 | 22.11 |
| Females | Population | 2.46 | 23.03 |
| Females | Basic | 2.20 | 22.17 |
| Females | Secondary | 2.63 | 23.24 |
| Females | Tertiary | 2.90 | 24.66 |

*Continued in the next page*

*D. Remaining Life Expectancy with Dementia*

| Sex | Education | start.Healthy_60 | start.Dem_60 |
| --- | --- | --- | --- |
| Males | Population | 1.10 | 10.10 |
| Males | Basic | 1.05 | 9.65 |
| Males | Secondary | 1.10 | 10.11 |
| Males | Tertiary | 1.23 | 11.08 |
| Females | Population | 1.89 | 13.47 |
| Females | Basic | 1.89 | 13.06 |
| Females | Secondary | 1.90 | 12.35 |
| Females | Tertiary | 1.94 | 14.43 |

*E. Remaining Life Expectancy with Comorbidity*

| Sex | Education | start.Healthy_60 | start.HL_60 | start.Dem_60 | start.Comorb_60 |
| --- | --- | --- | --- | --- | --- |
| Males | Population | 0.44 | 1.9 | 0.57 | 12.82 |
| Males | Basic | 0.4 | 1.84 | 0.58 | 12.08 |
| Males | Secondary | 0.46 | 1.89 | 0.61 | 13.13 |
| Males | Tertiary | 0.5 | 2.08 | 0.53 | 13.47 |
| Females | Population | 0.58 | 2.82 | 0.66 | 16.07 |
| Females | Basic | 0.55 | 2.82 | 0.67 | 15.45 |
| Females | Secondary | 0.61 | 2.87 | 0.71 | 15.84 |
| Females | Tertiary | 0.58 | 2.69 | 0.56 | 17.84 |

**Appendix 5.** Lifetime risk using different index ages.

| **Lifetime risk** | **Men** | **Women** |
| --- | --- | --- |
|  |  |  |
| *Index age = 70* |  |  |
| Lifetime risk of dementia (from healthy) | 24.60 | 33.53 |
| Lifetime risk of comorbidity (from hearing loss) | 35.91 | 44.24 |
| Lifetime risk of hearing loss (from healthy) | 20.72 | 20.26 |
| Lifetime risk of comorbidity (from healthy) | 8.63 | 9.73 |
|  |  |  |
| *Index age = 80* |  |  |
| Lifetime risk of dementia (from healthy) | 26.04 | 33.49 |
| Lifetime risk of comorbidity (from hearing loss) | 33.88 | 41.27 |
| Lifetime risk of hearing loss (from healthy) | 15.26 | 15.71 |
| Lifetime risk of comorbidity (from healthy) | 5.97 | 6.99 |
|  |  |  |
| *Index age = 90* |  |  |
| Lifetime risk of dementia (from healthy) | 16.13 | 20.97 |
| Lifetime risk of comorbidity (from hearing loss) | 18.48 | 23.52 |
| Lifetime risk of hearing loss (from healthy) | 8.46 | 8.31 |
| Lifetime risk of comorbidity (from healthy) | 1.98 | 2.23 |
|  |  |  |
| *Partial risk for ages 60-80, index age = 60* |  |  |
| Lifetime risk of dementia (from healthy) | 10.86 | 20.97 |
| Lifetime risk of comorbidity (from hearing loss) | 15.26 | 23.52 |
| Lifetime risk of hearing loss (from healthy) | 16.48 | 8.31 |
| Lifetime risk of comorbidity (from healthy) | 2.49 | 2.29 |
